# *Leishmania donovani* Triose Phosphate Isomerase: A Potential Vaccine Target against Visceral Leishmaniasis

**DOI:** 10.1371/journal.pone.0045766

**Published:** 2012-09-24

**Authors:** Pramod K. Kushawaha, Reema Gupta, Chandra Dev Pati Tripathi, Prashant Khare, Anil Kumar Jaiswal, Shyam Sundar, Anuradha Dube

**Affiliations:** 1 Division of Parasitology, Central Drug Research Institute, Lucknow, India; 2 Department of Medicine, Institute of Medical Sciences, Benaras Hindu University, Varanasi, India; Federal University of São Paulo, Brazil

## Abstract

Visceral leishmaniasis (VL) is one of the most important parasitic diseases with approximately 350 million people at risk. Due to the non availability of an ideal drug, development of a safe, effective, and affordable vaccine could be a solution for control and prevention of this disease. In this study, a potential Th1 stimulatory protein- Triose phosphate isomerase (TPI), a glycolytic enzyme, identified through proteomics from a fraction of *Leishmania donovani* soluble antigen ranging from 89.9–97.1 kDa, was assessed for its potential as a suitable vaccine candidate. The protein- *L. donovani* TPI (LdTPI) was cloned, expressed and purified which exhibited the homology of 99% with *L. infantum* TPI. The rLdTPI was further evaluated for its immunogenicity by lymphoproliferative response (LTT), nitric oxide (NO) production and estimation of cytokines in cured *Leishmania* patients/hamster. It elicited strong LTT response in cured patients as well as NO production in cured hamsters and stimulated remarkable Th1-type cellular responses including IFN-ã and IL-12 with extremely lower level of IL-10 in *Leishmania*-infected cured/exposed patients PBMCs *in vitro.* Vaccination with *LdTPI*-DNA construct protected naive golden hamsters from virulent *L. donovani* challenge unambiguously (∼90%). The vaccinated hamsters demonstrated a surge in IFN-ã, TNF-á and IL-12 levels but extreme down-regulation of IL-10 and IL-4 along with profound delayed type hypersensitivity and increased levels of *Leishmania*-specific IgG2 antibody. Thus, the results are suggestive of the protein having the potential of a strong candidate vaccine.

## Introduction

Visceral Leishmaniasis (VL) is a major public health problem among the poor population with significant morbidity and mortality worldwide wherein approximately 500,000 cases of VL are reported each year, 90% occur in Bangladesh, Brazil, India, Ethiopia, Kenya and Sudan, countries that are strongly related to poverty [1], [2], [3]. In India, high incidence has been reported from the states of Bihar, Assam, West Bengal and eastern Uttar Pradesh where resistance and relapse are on the increase [4]. With the advent of the HIV epidemics, the disease has emerged as an important opportunistic infection in AIDS patients [5], [6]. Available chemotherapy for VL is far from satisfactory because antileishmanial drugs are costly with unpleasant side effects. The situation has further worsened with emergence of drug resistance in various regions of endemicity [5]. Therefore, attention has now been shifted towards the development of effective vaccines. Although induction of lifelong protection against re-infection in recovered people demonstrates that a protective vaccine can be achieved, an effective vaccine against human leishmaniasis has yet to be discovered [7], [8], [9].

The characteristic clinico-pathological and immunological features of active VL is fever, hepatosplenomegaly, cachexia, pancytopenia, hypergammaglobulinemia and the absence of parasite-specific cell-mediated immune responses (CMI) [10], [11]. The major arms of CMI-Helper and cytotoxic T cells are known to play an integral role in the immune response to *Leishmania* infection, connecting the innate immune response to the development of efficient adaptive cellular immunity, mainly through IL-2 and IFN-γ production. These two cytokines drive the effector functions of macrophages and trigger a Th1 immune response [12]. Hence, any intervention that helps the shift of the immune response from Th2 type towards Th1 type will have a major role in cure and prevention of VL and therefore, modalities to immunopotentiate the Th1 arm of the immune response could be exploited as vaccine candidates. There are reports that protective immunity is achieved by upregulation of Th1 response after a successful chemotherapy [13], [14].

Based on this, recently, several potential protective antigens from *L. donovani* were identified through proteomics that induced a Th1 response in the PBMCs of cured/endemic *Leishmania* patients [15], [16]. Triose phosphate isomerase (TPI/TIM) is one of the vital glycolytic enzyme which has been identified as T- cell stimulatory protein. The protein is responsible for the reversible isomerisation between glyceraldehyde 3-phosphate (GAP) and dihydroxyacetone phosphate (DHAP). Due to its key role in energy metabolism, as well as its well-understood enzymatic and structural features, TPI of a pathogenetic organism is often considered as a potential drug and vaccine targets [17], [18]. The protein has been identified as a protective antigen in flat worm-*Schistosoma japonicum* (SjTPI) [19] and it has been demonstrated that a SjTPI DNA vaccine produced a 27.9–30.2% worm reduction in mice [20], [21].

In the present study we have cloned, expressed and purified *L.donovani* TPI (LdTPI) and assessed the ability of recombinant LdPTI (rLdTPI) to stimulate cellular response in the PBMCs of cured patients of VL and in mononuclear cells of cured *Leishmania* infected hamsters as well. The protein as a *LdTPI*-DNA was further evaluated for its prophylactic efficacy in golden hamsters (*Mesocricetus auratus*) which is a perfect experimental model for VL as it simulate the clinical profile of the disease in humans [22], [23].

## Materials and Methods

### Animal

Laboratory-bred male golden hamsters (*Mesocricetus auratus*, 45–50 g) from the Institute’s animal house facility were used as experimental host. They were housed in climatically controlled room and fed with standard rodent food pellet (Lipton India Ltd., Bombay) and water *ad libitum*. The usage of hamsters was approved by the Institute’s Animal Ethical Committee (protocol number 150/09/Para/IAEC dated 23.10.2009).

### Parasites

The *L. donovani* clinical strain 2001 was procured from a patient admitted to the Kala-azar Medical Research Centre of the Institute of Medical Sciences, BHU, Varanasi and was cultured *in vitro* as described elsewhere [24]. Promastigotes were grown in RPMI –1640 medium at 26°C (Sigma, USA) in 75 cm^2^ culture flasks (Nunc) [25], [26]. The strain has also been maintained in hamsters through serial passage, i.e. from amastigote to amastigote [25].

### Preparation of Soluble *L. donovani* Promastigote Antigen

Soluble *L. donovani* promastigote antigen (SLD) was prepared as per method described by Gupta et al. [15]. Briefly, log phase promastigotes (10^9^) were harvested from 3 to 4 days of culture and washed four times in cold phosphate-buffered saline (PBS) and resuspended in PBS containing protease inhibitors cocktail (Sigma, USA) and subjected to ultrasonication and centrifugation at 40,000×g for 30 min. The protein content of the supernatant was estimated by Lowry method [27] and stored at −70°C.

### Cloning, Expression and Purification of *L. donovani* Triose Phoaphate Isomeras (*LdTPI*)

RNA was isolated from 10^8^ promastigotes of *L. donovani*, by Trizol (Sigma) and cDNA was synthesized by Moloney murine leukemia virus reverse transcriptase as per the manufacturer’s protocol (Fermentas). Resulting cDNA was treated with DNase (10 µg/µl; Fermentas). *LdTPI* gene was amplified using Taq Polymerase (Takara) lacking a 3¢–5¢ exonuclease activity. PCR was performed using LdTPI-specific primers (based on the *L. infantum* - TPI gene sequence): forward, 5- GGATCCATGTCCGCCAAGCCCCAGCCGATC-3 and reverse, 5- GAATTCTCACTTCCGGGTGGCATCAATGATGTC-3 (*BamHI* and *EcoRI* site underlined) in a Thermocycler (Bio-Rad) under conditions at one cycle of 95°C for 2 min, 30 cycles of 95°C for 1 min, 54°C for 30 sec, and 72°C for 1 min, and finally one cycle of 72°C for 10 min. Amplified PCR product was electrophoresed in agarose gel and eluted from the gel by Gen Elute Columns (Qiagen). Eluted product was ligated in pTZ57R/T (T/A) cloning vector (Fermentas) and transformed into competent *Escherichia coli* (*E. coli*) DH5á cells. The transformants were screened for the presence of recombinant plasmids with the LdTPI insert by restriction digestion. Isolated positive clones were sequenced from DNA sequencing facility, Department of Biochemistry, South Campus- Delhi University (New Delhi), India and submitted to the NCBI http://www.ncbi.nlm.nih.gov/nuccore/EU867389.1 (accession no. EU920069.1). *LdTPI* was further sub-cloned at the *BamHI* and *EcoRI* site in bacterial pET28a vector (Novagen). The expression of *LdTPI* was checked in bacterial cells by transforming the *LdTPI* + pET28a construct in *E. coli* rosetta strain. The transformed cells were inoculated into 5-ml test tube Luria- Bertani medium (LB) and allowed to grow at 37°C in a shaker at 220 rpm. Cultures in logarithmic phase (at OD_600_ of ∼0.5–0.6) were induced for 15–18 h with 1.0 mM isopropyl-b-D-thiogalactopyranoside (IPTG) at 18°C. After induction, 1 ml cells were lysed in 100 µl sample buffer (50 mM Tris-HCl (pH 8), 10% glycerol, 10% SDS, and 0.05% bromophenol blue, with 100 mM DTT) and whole cell lysates (WCL) were analyzed by 12% SDS-PAGE. Uninduced control culture was analyzed in parallel. The over expression of rLdTPI was visualized by staining with coomassie – R250 stain (sigma).

Purification was carried out by taking 200 ml of LB medium containing 34 µg/mL of chloramphenicol and 35 µg/mL kanamycin, inoculated with *E. coli* rosetta strain transformed with pET28a + *LdTPI*, and grown at 37°C to an O.D._600_ of ∼0.6. Recombinant protein expression was induced by addition of 1 mM (IPTG,Sigma) and the culture was incubated at 18°C for an additional 15–18 h. The rLdTPI was purified by affinity chromatography using Ni^2+^-NTA chelating resin to bind the His6-tag fusion peptide derived from the pET28a vector. The cell pellet was resuspended in 4 ml of lysis buffer (50 mM Tris-HCL pH 8.0, 300 mM NaCl, and 20 mM imidazole,) containing 1∶200 dilution of protease cocktail inhibitor (Sigma) and 1% Triton X-100, incubated for 30 min on ice with 1 mg/ml lysozyme (Sigma), and the suspension was sonicated for 10×20 sec (with 30 s intervals between each pulse) on ice. The sonicated cells were centrifuged at 15,000 g for 30 min, and the supernatant was incubated at 4°C for 1 h with the 2 ml of Ni^2+^-NTA Superflow resin (Qiagen, Hilden, Germany) previously equilibrated with lysis buffer. After washing with buffer (50 mM Tris-HCL (pH 8.0), 300 mM NaCl and 1% Triton X- 100 or Triton X- 114 ), containing different concentrations of imidazole i.e. 20, 50 and 100 mM, the purified recombinant rLdTPI was eluted with elution buffer (50 mM Tris-HCL pH 7.5, 100 mM NaCl, and 250 mM imidazole). The eluted fractions were analyzed by 12% SDS–PAGE and the gels were stained with Coomassie brilliant blue R-250 (Sigma–Aldrich, St. Louis, USA). For *in vitro* experiments, imidazole from eluted protein was easily removed by dialysis using a 10 kDa molecular cut off dialysis device. The presence of endotoxin was tested by QCL-1000® Chromogenic LAL kit (Lonza). The protein content of the fractions was estimated by the Bradford method using bovine serum albumin (BSA) as standard.

### Production of Polyclonal Antibodies Against the rLdTPI and Western Blot Analysis

The purified rLdTPI protein was used for raising antibodies in New Zealand white rabbit. Rabbit was first immunized using 50 µg of rLdTPI in Freund’s complete adjuvant. After 15 days, the rabbit was given 3 booster doses of 25 µg rLdTPI each in incomplete Freund’s adjuvant at 2-weeks interval and blood was collected for serum 8 days after the last immunization. For immunoblotting experiment, purified rLdTPI protein and SLD were resolved on 12% SDS–PAGE and transferred on to nitrocellulose membrane using a semi-dry blot apparatus (Amersham) [28]. After overnight blocking in 5% BSA, the membrane was incubated with antiserum to the rLdTPI protein at a dilution of 1∶4000 for 120 min at room temperature (RT). The membrane was washed three times with PBS containing 0.5% Tween - 20 (PBS-T) and then incubated with goat anti-rabbit IgG HRP conjugate (Bangalore Genie) at a dilution of 1∶10,000 for1 h at room temperature. Blot was developed by using diaminobenzidine + imidazole +H_2_O_2_ (Sigma).

### Expression of *LdTPI*-DNA Construct in Mammalian Cell Line

The expression of *LdTPI*–DNA vaccine was further checked in mammalian cells by transfecting the *LdTPI*–DNA vaccine construct in HEK- 293T cells. For transfection and vaccination studies, endotoxin- free plasmid DNA was isolated using an Endofree plasmid purification Maxi kit as per the manufacturer’s protocol (Qiagen). For confirmation of the proteins expressed by the *LdTPI*–DNA vaccine construct, 2 x10^5^ HEK - 293T cells were grown in four-chamber slides and transfected with the blank pcDNA3 plasmid and *LdTPI*–DNA vaccine construct in serum-free DMEM (Life Technologies) using Lipofectamine 2000 (Invitrogen) according to the manufacturer’s protocol. Expression was confirmed by western blotting. For western blotting, the lysate of stably transfected HEK- 293T cells was prepared and subjected to SDS-PAGE. Thereafter, the protein bands were electrophoretically transferred to nitrocellulose membrane. To detect the expressed protein, a primary polyclonal antibody against rLdTPI was used at 1∶5000 dilution followed by 1∶10000 dilution of HRP-conjugated goat anti-rabbit IgG secondary antibody (Bangalore Genei, Bangalore, India) developed by using diaminobenzidine + imidazole +H_2_O_2_ (Sigma).

### Patients and Isolation of Peripheral Blood Mononuclear Cells (PBMCs)

The study was based on a convenience human sample of 27 belonging to the following categories/groups:

- [1] Ten treated cured patients (6 males and 4 females, age ranging from 5–40 years) from hyper-endemic areas of Bihar. All the patients had received complete course of Amphotericin B and had recovered from VL. Samples were collected from 2 months to 1 year after the completion of treatment. Diagnosis was established in all cases by demonstration of parasites in splenic aspirates and found negative at the time of study.
- [2] Seven endemic household contacts (3 females and 4 males, age range-15 to 45 years) that neither showed clinical symptoms nor received any treatment for Kala-azar.
- [3] Five infected patients (3 males and 2 females, age range- 5 to 45 years) showing clinical symptoms of Kala-azar.
- [4] Five normal healthy donors (3 males and 2 females, age range 25–35 years) from non-endemic areas, without any history of leishmaniasis, served as negative control.

The study was approved by the Ethics committee of the Kala-azar Medical Research Centre, Muzaffarpur (Protocol # EC-KAMRC/Vaccine/VL/2007–01). All the human subjects underwent clinical examination by a local physician for leishmanial and other possible infections. Informed consent was obtained from each subject before clinical examination and sampling.

Heparinized venous blood (10 ml each) was collected from all the study subjects and peripheral blood mononuclear cells (PBMCs) were isolated from blood by Ficoll Hypaque density gradient centrifugation (Histopaque 1077, Sigma, USA) as described by Garg et al. [24]. A final suspension of 1×10^6^ cells/ml was made in complete RPMI medium (cRPMI) after determining cell viability by trypan blue staining method.

### Treatment of *L. donovani* Infected Hamsters and Isolation of Mononuclear Cells (Lymph Node Cells)

Approximately 20 hamsters, infected with 10^7^ amastigotes intracardially, were assessed one month later for parasitic burden by splenic biopsy through a small incision in the upper left quarter of the abdomen and a small piece of splenic tissue was cut and dab smears were made on slides. The incised portion was stitched with nylon suturing thread. Following biopsy, an adequate amount of antibiotic powder (Neosporin) was applied on the stitched portion and finally sealed with tincture of benzoin. In addition, neosporin sulphate (100 mg/kg of body weight) was also given orally the day before and the day after the biopsy for healing. The smears were fixed in methanol and stained with Giemsa stain and the number of amastigotes/1000 cell nuclei was counted. The animals harbouring >25–30 amastigotes/100 macrophage cell nuclei were then treated with antileishmanial drug-Miltefosine (Zentaris, Germany) at 40 mg/kg bodyweight daily for 5 days. The animals were reassessed for complete cure by splenic biopsy performed on day 30 post-treatment. Mononuclear cells were separated from lymph nodes of cured, infected as well as normal hamsters following the protocol of Garg et al. [24] and a suspension of 10^6^ cells/ml was made in cRPMI. These cells were employed for lymphoproliferative assay and for the estimation of NO production.

### Assessment of Prophylactic Efficacy of *LdTPI*-DNA Vaccine in Hamsters to Leishmania Challenge

Four experimental groups each having 12–15 hamsters were taken for the study. The hamsters of Group I was immunized intramuscularly (i.m.) with *LdTPI*-DNA (100 µg/100 µl PBS/animal) alone whereas groups II to IV served as controls. The animals of group II were vaccinated with 100 µg of pcDNA3 (blank vector)/100 µl of PBS and that of group III served as unvaccinated but infected control. The animals of group IV did not receive either DNA vaccine or *Leishmania* infection and served as normal control. Two booster doses of 100 µg *LdTPI*-DNA were given i.m. to group I on day 14 and 21. Day twenty-eight days after the first vaccination dose, all the vaccinated and unvaccinated control groups were challenged intracardially with 100 µl of 10^8^ cell/ml late log phase promastigotes (10^7^) of *L. donovani*, isolated on day 3–4 of culture which contains mostly late log phase or stationary or metacyclic forms. The body weight and that of the spleen and liver (on necropsy) of hamsters of all of the experimental groups were assessed, on necropsy, at different time intervals, i.e., on days 0, 45, 90 and 120 post-challenge (p.c.). The assessment of parasite burden was done in spleen, liver and bone marrow of vaccinated hamsters at different time intervals, i.e. on days 0, 45, 90, 120 p.c. Peritoneal exudates cells, inguinal lymph nodes and blood were also collected at these time points to obtain cells and sera for evaluation of cellular and antibody responses as per protocols described below. The criterion for prophylactic efficacy was the assessment of parasite load as the number of amastigotes/1000 cell nuclei in each organ in comparison to the unvaccinated controls and the percentage inhibition (PI) was assessed in comparison to the unvaccinated control by following formula [29] PI  =  (No. of parasite count from infected control −No. of parasite from vaccinated group/No. of parasite count from infected control) × 100. DTH was performed by injecting 50 µg/50 µl of SLD in PBS intradermally into one footpad and PBS alone into the other one of each of the vaccinated and unvaccinated controls. The response was evaluated 48 h later by measuring the difference in footpad swelling between the two with and without SLD for each animal [30].

### Immunological Assays

#### Assessment of Lymphocyte proliferative responses (LTT) in cured/exposed patients and hamsters

PBMCs and mononuclear cells (1×10^6^ cells/ml) of cured/exposed patients and normal, infected (30 days p.c.) as well as cured hamsters respectively, was cultured in 96-well flat bottom tissue culture plates (Nunc, Denmark). This assay was carried out as per protocol described by Kushawaha et al. [31]. About 100 µl of predetermined concentration of mitogens phytohaemoglutinin (PHA, 10 µg/ml Sigma, USA) for Patient’s PBMCs, Concavalin A (Con A, 10 µg/ml, Sigma, USA) for hamster’s mononuclear cells, as well as rLdTPI and SLD (10 µg/ml each) were added to the wells in triplicate. Wells without stimulants served as blank controls. Cultures were incubated at 37°C in a CO_2_ incubator with 5% CO_2_ for 3 days in the case of the mitogens, and for 5 days in the case of the antigens. Eighteen hours prior to termination of experiment, 50 µl of XTT (Roche diagnostics) was added to 100 µl of supernatants of each well and absorbance measured at 480 nm with 650 nm as reference wavelength.

#### Estimation of Nitric oxide (NO) activity in macrophages of hamsters

Isolated mononuclear cells from all the three study groups of hamsters viz. Normal, infected (30 post infected) and cured, were suspended in culture medium and plated at 10^5^ cells/well and stimulated for 3 days in case of mitogen (LPS, 10 µg/ml) and 5 days in case of antigens (rLdTPI, SLD) at 10 µg/ml. The presence of nitric oxide (NO) was assessed using Griess reagent (Sigma, U.S.A) in the culture supernatants of naive hamster peritoneal macrophages [24] after the exposure with 100 µl supernatant of stimulated mononuclear cells. The supernatants (100 µl) collected from macrophage cultures 24 h after incubation at 37°C in CO_2_ incubator was mixed with an equal volume of Griess reagent (Sigma, USA) and left for 10 min at room temperature. The absorbance of the reaction was measured at 540 nm in an ELISA reader [32]. The nitrite concentration in the macrophages culture supernatant samples was extrapolated from the standard curve plotted with sodium nitrite. The same protocol for measuring NO production was used in *LdTPI*-DNA vaccination study.

#### Assessment of Cytokine levels- IFN-ã/IL-12/IL-10 in PBMCs of cured/endemic patients

Culture of PBMCs (1×10^6^ cells/ml) from human patients was set up in 96-well culture plates and rLdTPI as well as SLD were added in triplicate wells at a concentration of 10 µg/ml. The level of IFN-ã, IL-12 and IL-10 was estimated by enzyme-linked immuno-sorbent assay (ELISA) kit (OptEIA set, Pharmingen) after 5 days of incubation with antigen using supernatants. The results were expressed as pictograms (pg) of cytokine/ml, based on the standard curves of the respective cytokine provided in the kit. The lower detection limits for various cytokines were as follows: for IFN-ã- 4.7 pg/ml, for IL-12p40- 7.8 pg/ml and for IL-10 −7.0 pg/ml.

#### Quantification of mRNA cytokines and inducible NO synthase (iNOS) in hamsters by Real time-PCR (QRT-PCR)

QRT-PCR was performed to assess the expression of mRNAs for various cytokines and iNOS in splenic cells. Splenic tissues were taken from each of the three randomly chosen animals. Total RNA was isolated using Tri-reagent (Sigma-Aldrich) and quantified by using Gene-quant (Bio-Rad). One microgram of total RNA was used for the synthesis of cDNA using a first-strand cDNA synthesis kit (Fermentas). For qRT- PCR, primers were designed using Beacon Designer software (Bio-Rad) on the basis of cytokines and iNOS mRNA sequences available on PubMed [22] (Table 1). qRT-PCR was conducted as per the protocol described earlier by Kushawaha et al. [31]. Briefly, it was carried out with 12.5 µl of SYBR green PCR master mix (Bio-Rad), 1 µg of cDNA, and primers at a final concentration of 300 nM in a final volume of 25 µl. PCR was conducted under the following conditions: initial denaturation at 95°C for 2 min followed by 40 cycles, each consisting of denaturation at 95°C for 30 s, annealing at 55°C for 40 s, and extension at 72°C for 40 s per cycle using the iQ5 multicolor real-time PCR system (Bio-Rad). cDNAs from infected hamsters were used as “comparator samples” for quantification of those corresponding to test samples whereas in vaccination studies. All quantifications were normalized to the housekeeping gene HPRT. A no-template control cDNA was included to eliminate contaminations or nonspecific reactions. The cycle threshold (CT) value was defined as the number of PCR cycles required for the fluorescence signal to exceed the detection threshold value (background noise). Differences in gene expression were calculated by the comparative CT method [33]. This method compares test samples to a comparator sample and uses results obtained with a uniformly expressed control gene (HPRT) to correct for differences in the amounts of RNA present in the two samples being compared to generate a ΔCT value. Results are expressed as the degrees of difference between ΔCT values of test and comparator samples.

**Table 1:** Sequences of forward and reverse primers of hamster cytokines used for quantitative real time -PCR.

S.N.	Primer	Primer Sequence
1	HPRT	Forward 5′ GATAGATCCACTCCCATAACTG 3′
		Reverse 5¢ TACCTTCAACAATCAAGACATTC 3′
2	TNF-á	Forward 5¢ TTCTCCTTCCTGCTTGTG 3¢
		Reverse 5′ CTGAGTGTGAGTGTCTGG 3′
3	IFN-ã	Forward 5′ GCTTAGATGTCGTGAATGG 3′
		Reverse 5¢ GCTGCTGTTGAAGAAGTTAG 3′
4	IL-12	Forward 5′ TATGTTGTAGAGGTGGACTG 3′
		Reverse 5′ TTGTGGCAGGTGTATTGG 3′
5	TGF-â	Forward 5′ ACGGAGAAGAACTGCTGTG 3′
		Reverse 5′ GGTTGTGTTGGTTGTAGAGG 3′
6	IL-4	Forward 5′ GCCATCCTGCTCTGCCTTC 3′
		Reverse 5′ TCCGTGGAGTTCTTCCTTGC 3′
7	IL-10	Forward 5′ TGCCAAACCTTATCAGAAATG3’
		Reverse 5′ AGTTATCCTTCACCTGTTCC 3′
8	iNOS	Forward 5′ CGACGGCACCATCAGAGG 3¢
		Reverse 5′AGGATCAGAGGCAGCACATC 3′

#### Measurement of antibody response in hamsters

The level of IgG antibody and its isotypes in sera samples of hamsters of different experimental groups was measured as per protocol described by Kushawaha et al. [31]. Briefly, 96-well ELISA plates (Nunc) were coated with SLD (0.2 µg/100 µl/well) overnight at 4°C and blocked with 1.5% BSA at room temperature for 1 h. Sera was used at a dilution of 1/100 for IgG, IgG1, and IgG2 and kept for 2 h at room temperature. Biotin-conjugated mouse anti-Armenian and Syrian hamster IgG, IgG1 and biotinylated anti-Syrian hamster IgG2 (BD Pharmingen) were added for 1 h at room temperature at 1/1000 dilutions and were further incubated with streptavidin-conjugated peroxidase at 1/1000 (BD Pharmingen) for 1 h. Finally, the substrate O-phenylenediamine dihydrochloride (Sigma-Aldrich) was added and the plate was read at 492 nm.

### Post-challenge Survival

Survival of hamsters belonging to group I was checked until day 180 p.c. in comparison to the normal hamsters. Animals in all of the groups were given proper care and were observed for their physical conditions until their survival period. Survivals of individual hamsters were recorded and mean survival period was calculated.

### Statistical Analysis

Results were expressed as mean±S.D. In each experiment 12–15 animals were used in each group. Two sets of experiments were performed and the results were analyzed by one-way ANOVA test followed by Dunnets or Tukeys post which ever appropriate at each case using Prism Graphpad software program. The upper level of significance was chosen as p<0.001 (highly significant).

## Results

### *LdTPI* was Cloned, Sequenced, Expressed in *E. coli* Rosetta Strain and HEK Cell Line

**Figure 1:**
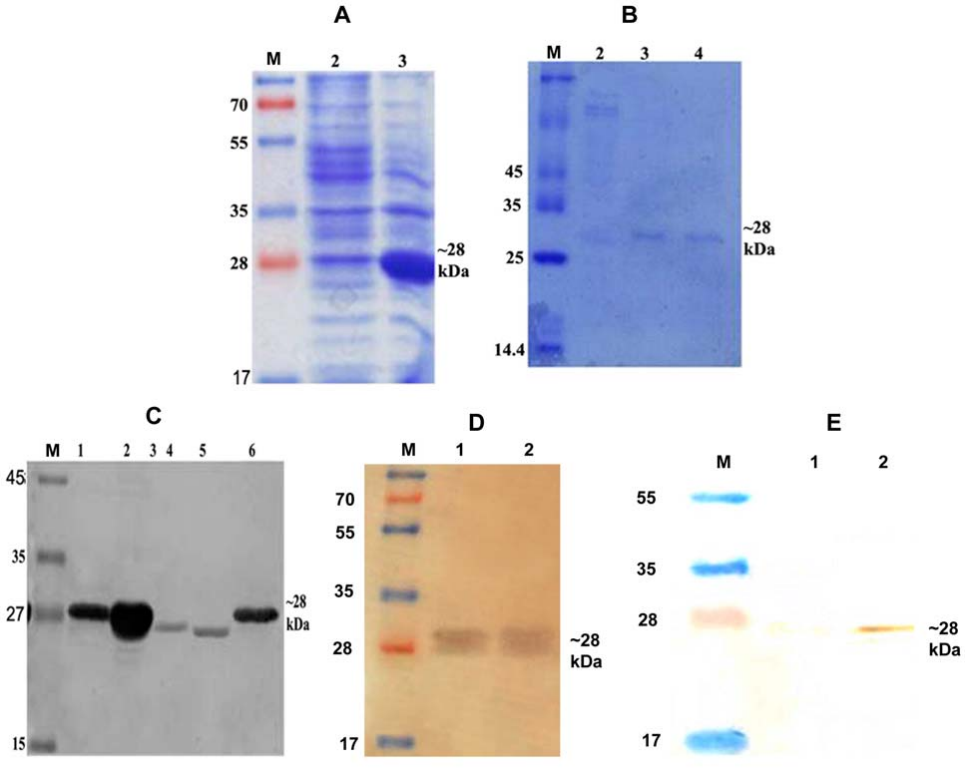
Cloning, expression and purification LdTPI. Expression of LdTPI in prokaryotic cells, whole cell lysate (WCL) of pET 28a+*LdTPI* transformed *E. coli* separated on 12% SDS-PAGE and stained with Coomassie brilliant blue, M: Molecular wt. markers; Lane 1: WCL before IPTG induction, lane 2: WCL after IPTG (1.0 mM) induction at 18°C (A); Purification of rLdTPI, M: Molecular wt. markers, Lane 1: wash fraction containing nonspecific proteins also and Lane 3 & 4: eluted purified protein (B); Western blot analysis using anti-LdTPI antibody raised in rabbit, M: Molecular wt. Markers, Lane 1: uninduced WCL of E. coli, Lane 2: induced WCL of E. coli, Lane 3&4: WCL and SLD of *L. donovani*, and Lane 5: purified rLdTPI (C); Western blot analysis of rLdTPI with patients’ serum. M: Molecular wt markers, Lane 1&2 blot with rLdTPI (D). Confirmation of LdTPI expression in HEK cell line by western blotting. M: Molecular wt. markers, Lane 1: WCL of non transfected cells, Lane 2: WCL of LdTPI-DNA construct transfected cells (E).

The *LdTPI* gene of *L. donovani* was successfully cloned and sequenced which was 99% homologous with *L. infantum* TPI (Table 2). Further it was expressed and purified by affinity chromatography using Ni^2+^ NTA coloums. The expression revealed that the size of the expressed proteins was ∼ 28 kDa (Fig. 1 A). The rLdTPI protein was purified and eluted at 250 mM imidazole concentration (Fig. 1 B). Immunoblots of lysates from *L. donovani* promastigotes was performed with the polyclonal anti-rLdTPI antibody which detected one dominant protein species of ∼27 kDa (Fig. 1 C). Western blot analysis of rLdTPI with patients’ sera also showed a dominant protein band of ∼28 kDa. The expression of rLdTPI in HEK cell line was confirmed by western blotting using anti-rLdTPI antibody (Fig. 1 D). The results showed that the recombinants were correctly constructed and expressed in mammalian cell line.

**Table 2:** Homology of LdTPI of *L. donovani* with other *Leishmania* species.

Species	Homology (%)
*Leishmania infantum*	99
*Leishmania major*	94
*Leishmania braziliensis*	84

### *rLdTPI* Stimulation Induced Strong Lymphoproliferative and NO Responses in Cured Hamsters

**Figure 2:**
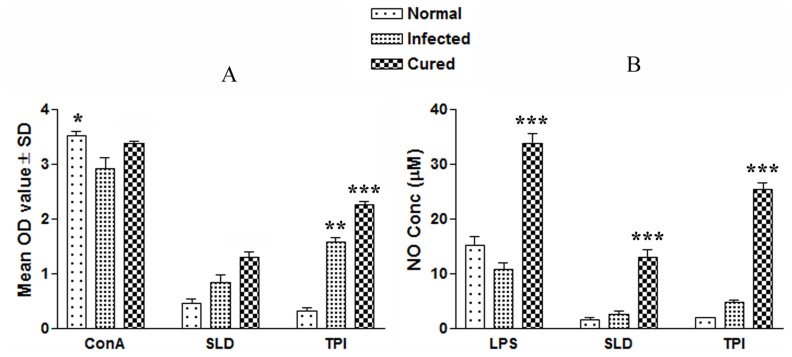
Cellular responses of rLdTPI in hamsters. LTT response of mononuclear cells (lymph nodes) from normal, *L. donovani* infected (30 day p.c.) and treated hamsters in response to Con A, SLD and rLdTPI at 10 µg/ml each. Proliferation was represented as mean O.D of stimulated culture - mean O.D of unstimulated control. Each bar represents the pooled data (mean ± S.D. value) of 6 hamsters and the data represent the means of triplicate wells ± S.D. of each hamster (A); Nitric oxide production (µM) by peritoneal macrophages of hamsters (n  = 5). The peritoneal macrophages were primed with 100 µl supernatants of stimulated mononuclear cells (3 days with mitogen and 5 days with antigens) of normal, infected and cured hamsters in response to rLdTPI, SLD and LPS respectively at 10 µg/ml each (B); The estimation of NO production was done using Greiss reagent in supernatants collected from macrophage cultures 24 h after incubation and the absorbance of the reaction product was measured at 540 nm. Significance values indicate the difference between the SLD and rLdTPI stimulation (*, p<0.05; **, p<0.01; and ***, p<0.001).

The cellular responses of lymph node cells of cured hamsters were assessed by XTT against the mitogen, i.e. Con A as well as SLD and rLdTPI. The responses were compared with that of normal as well as *L. donovani* infected groups that served as controls. Concavalin A controls showed a good lymphoproliferative (LTT) response in normal, active VL and cured groups, indicating the procedural sensitivity. The results of the proliferative response of mononuclear cells against rLdTPI showed considerably higher stimulation in cured hamsters (mean OD- 2.259±0.038) than infected hamsters (1.578±0.193). The difference was statistically significant (P<0.001) (Fig. 2 A).

NO is the critical killing effector molecule against leishmaniasis produced by IFN-ã stimulated and inducible NO synthase induced classical macrophages, hence its production in peritoneal macrophages of cured hamsters, was studied after 24 h of incubation in the presence of rLdTPI and SLD. For comparison, NO production in mitogenic (LPS) stimulated and unstimulated cells served as positive and negative controls respectively. NO production was recorded to be higher against rLdTPI (p<.001) (Fig. 2 B).

### *rLdTPI* Stimulates PBMCs from *Leishmania* Infected Cured/endemic to Proliferate and to Express a Robust Thl Cytokine Profile

**Figure 3:**
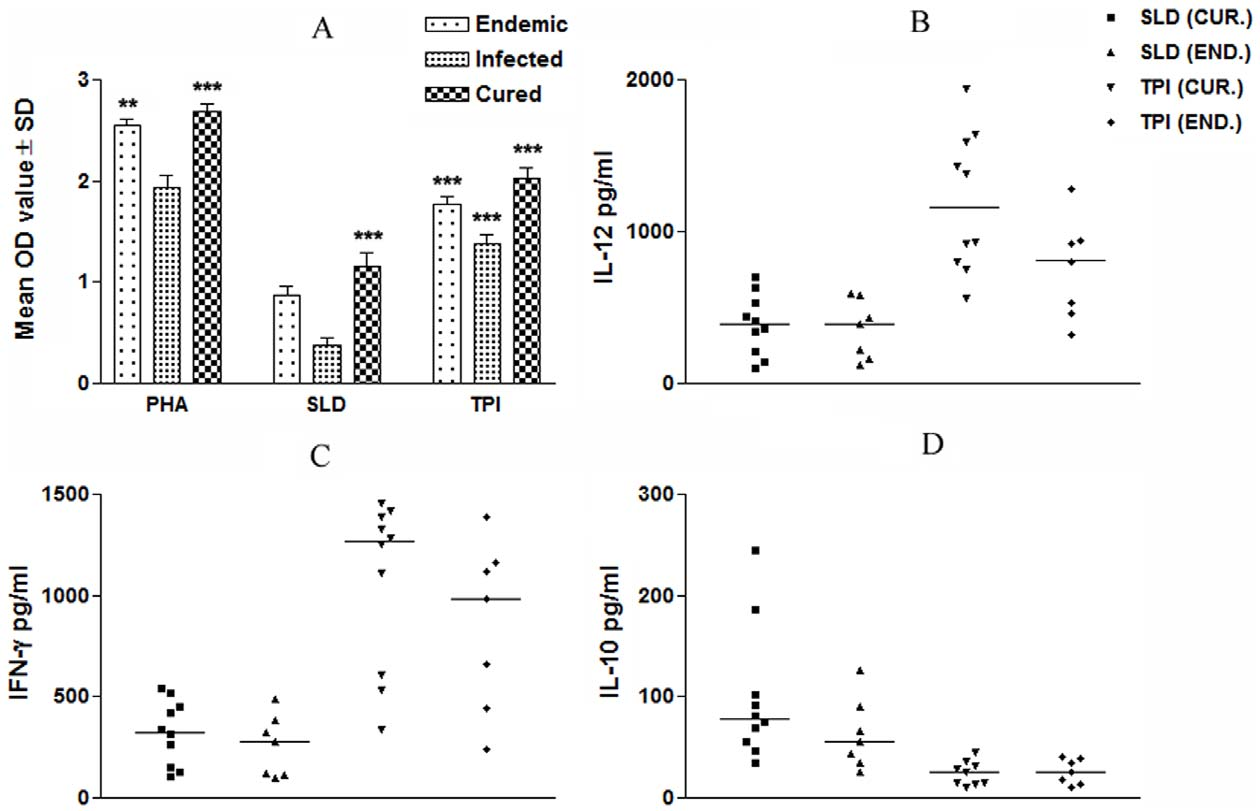
Cellular responses of LdTPI in human samples. LTT response, proliferation was represented as mean O.D of stimulated culture - mean O.D of unstimulated control. Each bar represents the pooled data (mean ± S.D. value) of stimulated PBMCs of each group (A); Th1 and Th2 cytokine production in PBMCs from individuals of cured VL patients (n = 7) and endemic controls (n = 5) in response to rLdTPI and SLD antigens, each data point represents one individual (B-D). Values are given as concentration in pg/ml. The statistical significances are given between infected vs cured and infected vs endemic individuals. (*, p<0.05; **, p<0.01; and ***, p<0.001).

We further validated the cellular responses (LTT and cytokine levels) of rLdTPI in PBMCs of cured patients, endemic and *L. donovani*-infected donors. Individual donors in each study group were found to elicit different responses. Proliferation and cytokine responses of PBMCs from patients with active VL/cured/endemic were compared using rLdTPI and SLD. Endemic control, cured and infected patients exhibited good proliferation against PHA, indicating the procedural sensivity. PBMCs from all the cured and active VL patients proliferated in response to rLdTPI with mean OD values of 2.019±0.103 and 1.378±0.123 higher values than SLD (mean OD values of 1.158±0.335) and 0.3673±0.118) respectively (p<.001) (Fig. 3 A).

To assess the Th1/Th2 stimulatory potential of the LdTPI we further studied the cytokine levels viz. IFN-ã and IL-12 in PBMCs from cured/infected patients as well as in endemic contacts against rLdTPI. The levels of IL-12 and IFN-γ were observed to be higher in the supernatants of cured patients with a range of 565.34–1945.75 pg/ml and 338.28–1458.68 pg/ml respectively as compared to endemic contacts (459.07–1280.87 pg/ml and 243.63–1385.13 pg/ml respectively). On the contrary, very low level of IL-10 cytokines against rLdTPI was detected in supernatants of cured (10.69–44.52 pg/ml) patients followed by endemic contacts (ranging from 9.3–40.23 pg/ml). PBMCs of cured/endemic contacts generated a mixed Th1/Th2 cytokine profile against SLD wherein high levels of IL-10 very little level of IFN-γ, and IL-12p40 were noticed in response to SLD in cured/endemic contacts(Fig. 3 B-D). We have not able to detect lymphoproliferative response and these cytokines in non endemic and infected control against rLdTPI.

### Vaccination with *LdTPI*-DNA Vaccine Induced Outstanding Protection in Hamsters against *L. donovani*-challenges

**Figure 4:**
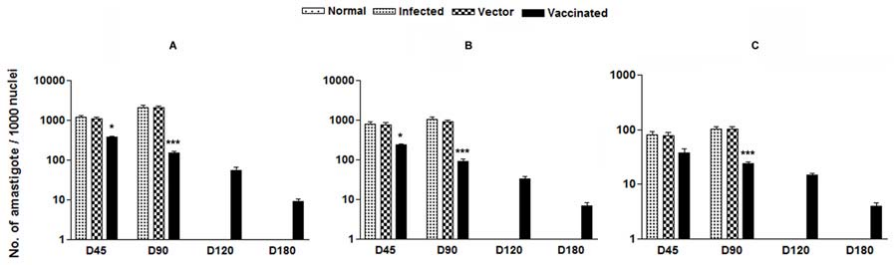
Parasite burden (no. of amastigotes per 1000 cell nuclei). Parasite load in the spleen (A), liver (B), and bone marrow (C) of infected control, vector (pcDNA) control as well as *LdTPI*-DNA vaccinated hamsters on days 0, 45, 90, 120, and 180 p.c. Significance values indicate the difference between the vaccinated groups and infected group (p<0.05; p<0.01; and p<0.001).

The ability of rLdTPI to stimulate T cell response and Th1 profile suggested that vaccintion with LdTPI may provide protection in *L. donovani*-infected hamsters. The *LdTPI*-DNA vaccinated hamsters were found to be optimally protected (∼90%) from the challenge infection of *L. donovani*. An increase from 10^3^ to 10^4^ parasites in all of the groups, except in the *LdTPI*-DNA vaccinated group, was seen in Giemsa-stained splenic smears from days 45 to 120 p.c. In the vaccinated group, parasite loads decreased from 2×10^2^ on day 45 to a very low level (p<0.001) by day 120 p.c. Similarly, in liver and bone marrow, parasite loads decreased sharply after day 45 p.c. and parasites were almost absent by day 180 p.c. in the vaccinated group (Fig.4 A-C). Cultivation of the spleen, liver, and lymph node tissues from the vaccinated hamsters *in vitro* yielded no promastigotes after prolonged incubation for 3 week. The *LdTPI*-DNA vaccinated hamsters survived the challenges of *L. donovani* and remained healthy more than 8 months. In contrast, hamsters vaccinated with the pcDNA3 vector and infected control survived for only 3–4 months.

### *LdTPI*-DNA Vaccination Stimulates DTH, Mitogenic and *Leishmania*-specific Cellular Responses

**Figure 5:**
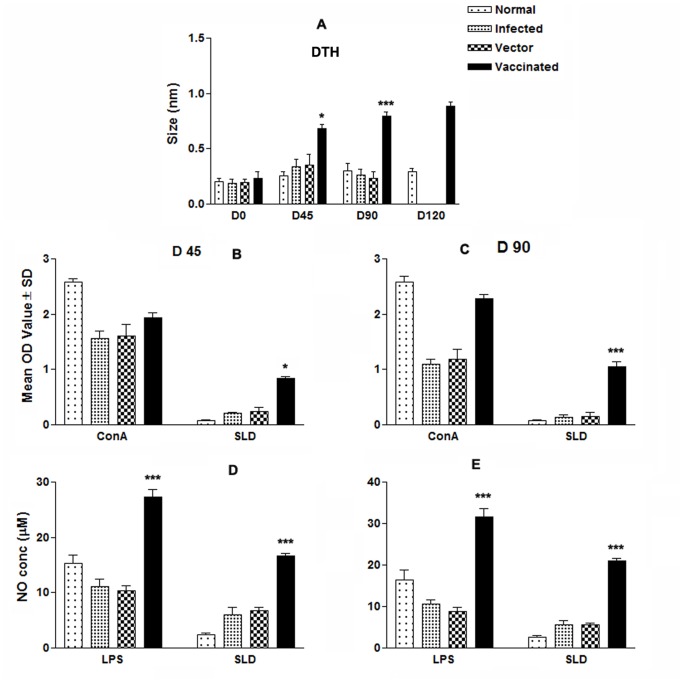
Cellular response against SLD in normal, infected control, vector (pcDNA) control as well as LdTPI-DNA vaccinated hamsters at different time intervals post challenge. (A) DTH response (mm); (B&C) LTT response (mean O.D value) to SLD and ConA in normal, infected control, vector (pcDNA) control as well as *LdTPI*-DNA vaccinated hamsters on days 45 and 90 post challenge; (D&E) NO production (µM) to LPS and SLD in the naive macrophages co-incubated with supernatants of mononuclear cells isolated from *LdTPI*-DNA-vaccinated hamsters in comparison to the unimmunized infected controls, vector-immunized controls, and uninfected normal hamsters on day 45 and day 90 post challenge. The *LdTPI*-DNA vaccinated hamsters survived the challenges of *L. donovani* and remained healthy more than 8 months. In contrast, hamsters vaccinated with the pcDNA3 vector and infected control survived for day 90 p.c. Significance values indicate the difference between the vaccinated groups and infected group (*, p<0.05; **, p<0.01; and ***, p<0.001).

DTH, an index of cell mediated immunity *in vivo*, and an Ag specific *in vitro* T cell proliferation assay revealed the status of cellular responses generated in vaccinated animals. We were therefore interested to see the DTH and proliferative responses elicited by vaccinated and challenged animals. Challenge with *L. donovani* induced enhanced DTH responses and LdTPI-specific T cell proliferation in all vaccinated hamsters at different time periods as compared to blank vector and infected controls. *LdTPI*-DNA vaccinated hamsters displayed significant DTH responses, which increased progressively and was higher than those of the control groups (p<0.01) at all time points for the duration of the experiments for up to 120 days (Fig.5 A). *In vitro* stimulation of the mononuclear cells with Con A showed comparable proliferative responses at high levels in all of the groups when assayed before challenges (day 0). ConA induced LTT response remained elevated in *LdTPI*-DNA vaccinated animals as much as those of the normal hamsters throughout the entire post challenged period, but it decreased precipitously with time in all of the other control groups. In SLD-specific re-stimulation assays, the lymphoproliferative response was negative for all the groups on pre-vaccination and for the non vaccinated control groups throughout the p.c. period (normal, infected and vector). Cells from *LdTPI*-DNA vaccinated hamsters produced a significantly higher response (p<0.001), which reached almost to the maximum on day 90 (Fig.5 B&C).

Lymphocyte-mediated activation of macrophages to produce NO for leishmanicidal activities was found to differ between control and experimental groups of hamsters. Supernatants from stimulated mononuclear cells of hamsters vaccinated with the *LdTPI*-DNA, when incubated with naive macrophages produced significant (p<0.001) amounts of nitrite (24 µg)which was ∼5 fold more than that of unimmunized infected controls and ∼8-fold more than the normal control group on day 90 p.c. (Fig.5 D&E).

### *LdTPI*-DNA Vaccination Favours the Development of Th1-type Cytokine Profile as Determined by Quantitative Real-time PCR

**Figure 6:**
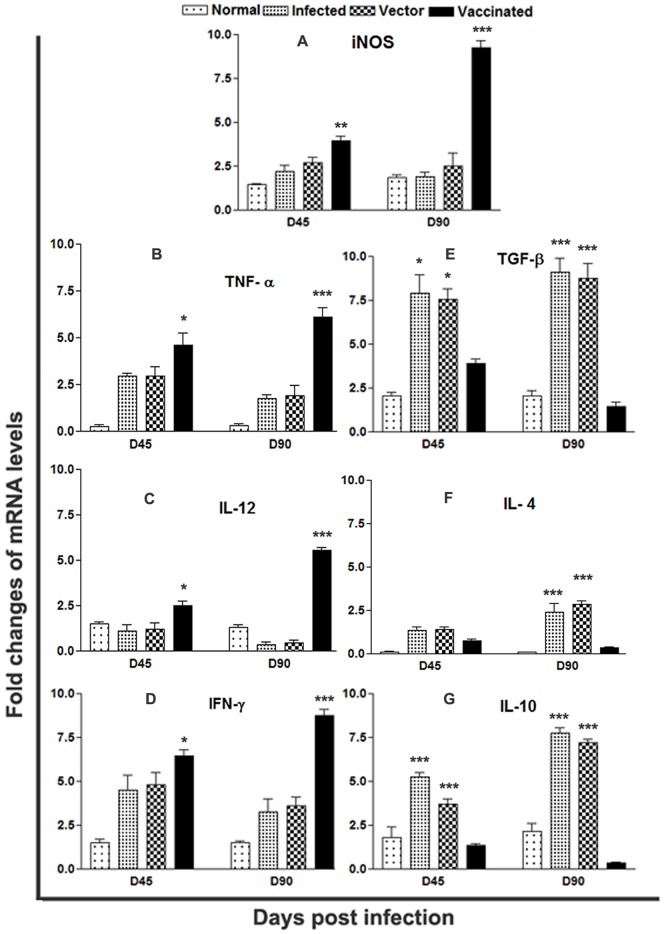
Splenic iNOS and cytokine mRNA expression profile analysis of normal and LdTPI-DNA vaccinated hamsters on days 45 and 90 p.c. by quantitative real-time PCR. Significance values indicate the difference between the vaccinated group and infected group (*, p<0.05; **, p<0.01; and ***, p<0.001).

It is well established that the cytokine milieu at the initiation of infection is critical in determining disease outcome [34], [35]. Since LdTPI elicited Th1 response in patients’ PBMCs, we further examined the cellular immune responses in *LdTPI* -DNA vaccinated hamsters. The expression of Th1 and Th2 mRNA cytokines was evaluated by real-time PCR on days 45 and 90 p.c. The expression of iNOS transcripts was observed to be significantly (p<0.001,) elevated by ∼ 4 fold in *LdTPI* -DNA vaccinated hamsters on day 90 p.c. (Fig.6 A). Similarly, at the same time point, the expression of TNF-á was also significantly higher (p<0.001) by ∼ 3 fold in the vaccinated group in comparison to *L. donovani*-infected group (Fig.6 B). The expression of IL-12 which was least expressed in the infected group on day 90 p.c. but was significantly (p<0.001) expressed by ∼5 fold higher in vaccinated hamsters on days 90 p.c (Fig.6 C). IFN-γ was suppressed in the infected group on day 45and 90.p.c, but was significantly higher by ∼ 3 fold in the vaccinated group on day90 (p<0.001) p.c. (Fig.6 D). The level of IL-10, IL-4 and TGF-â were significantly (p<0.001) up-regulated in infected and vector control, indicating progressive VL at different time intervals while these cytokines were highly down regulated in vaccinated hamsters reflecting the situation in cured VL (Fig.6 E–G).

### *LdTPI*-DNA Vaccination alters *Leishmania*-specific IgG and its Isotypes

Since the outcome of VL may be determined by the extent of immune system activation, it was highly important to characterize the changes in the immunoglobulin level after immunization. It is well established that the cytokines such as IFN-ã and IL-4 direct immunoglobulin class switching of IgG2a and IgG1, respectively.

**Figure 7:**
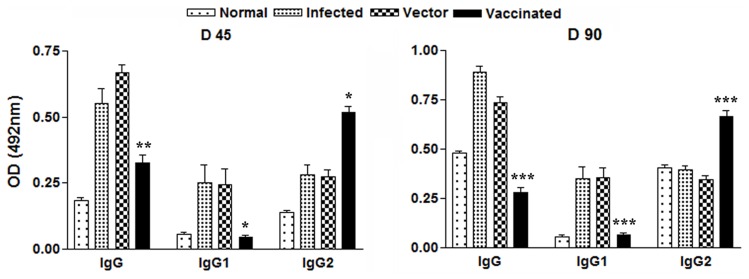
Anti- Leishmania-specific IgG and its isotypes IgG1 and IgG2 in LdTPI-DNA vaccinated hamsters in comparison to the unimmunized infected hamsters on days 45 and 90 p.c. Significance values indicate the difference between the vaccinated group and infected group (*, p<0.05; **, p<0.01; and ***, p<0.001).

The anti-Leishmania IgG and IgG1, a surrogate marker of Th2 cell differentiation were elevated progressively with time to a high level in all groups, except the *LdTPI*-DNA vaccinated, in which case they remained essentially in the background levels of the nonimmunized and unchallenged normal and vector immunized. In contrast, *LdTPI*-DNA vaccinated animals were the only group that showed a significant elevation by 2- to 3 fold over the others (p<0.01) in the level of IgG2, a surrogste marker for Th1 type immune response (Fig. 7). As a measure of CMI, the elevation of IgG2 was consistent with the development of effective immune responses.

## Discussion

It is established through clinical studies that human VL has a complex immunology characterized by mixed T-helper-1(Th1)/T-helper-2 (Th2) cell milieu, where a suppressed Th1 response along with an elevated Th2 is the hallmark of the active disease. On the other hand, protective immunity is achieved by upregulation of Th1 response after a successful chemotherapy [13]. Recovery from VL is always associated with immunity to subsequent infection and induction of Th1 cytokines, dominated by IFN-γ, which activate macrophages to kill the intracellular organisms, primarily through H_2_O_2_- dependent [36] and NO-mediated mechanism [37], [38]. Therefore, the antigens that are involved in the induction and recall of such memory Th1 cells are considered to be of great interest in the vaccine design strategy against VL.

A large number of leishmanial antigens against experimental leishmaniasis have been attempted for vaccination, mainly against the cutaneous form. However, there is still only a little progress with a limited number of potential candidates to combat VL through vaccination [39], [40]. During the last decades there are reports of defined antigens protective against experimental VL such as H2A/H2B, LelF-2, KMP11, HASPB, A2, ORFF, LACK and CPB [31], [41], [42], [43], [44], [45], [46], which have been found to be either partially protective or yet at the stage of experimental evaluation [47], [48], [49]. So far successful immunization trials were done with viz FML, FML-QuilA Saponin etc against canine visceral leishmaniasis (14–19). However, as compared to defined single antigen, polyprotein vaccines viz. LEISH-F1 (formerlyLeish111f), consisting of three recombinant antigens: *Leishmania* elongation initiation factor (LeIF), thiol-specific antioxidant (TSA) and *L. major* stress-inducibleprotein-1(LmSTI1) in combination with MPL-SE have been reported to afford better protection against experimental VL. This vaccine is the only one that has successfully cleared the clinical trial for safety and immunogenicity against leishmaniasis [50], [51] and is still under clinical efficacy trial. Hence, there is a need for identifying new antigens from *L. donovani* ideally relying on correlates of protective immunity.

On the basis of the fact that recovery from VL is always associated with immunity to subsequent infection and induction of Th1 cytokines dominated by IFN-γ, we identified several Th1 stimulatory proteins from soluble fraction and sub-fraction of *L. donovani* ranging from 89.9 to 97.1 kDa through proteomics which was also found to be protective against experimental VL [15], [16], [52]. Triose phosphate isomerase of *L. donovani* (LdTPI), a vital glycolytic enzyme, was one such Th1 stimulatory protein identified from the above stated fraction of soluble Leishmania antigen. In this study, we have re-assessed LdTPI for its possible immunogenic and prophylactic potential against VL. LdTPI, which was cloned, expressed and purified, has the homology with *L. infantum* TPI to the tune of 99%. Immunoblot study of *L.donovani* promastigote lysate with the polyclonal anti-rLdTPI antibody revealed one dominant protein of ∼27 kDa. The presence of this protein in higher molecular weight range in proteomic studies, in contrast to its observed molecular mass could be attributed to the post-translational modifications which are widely prevalent in *Leishmania.*

When evaluated for its immunogenicity by LTT and cytokine responses in PBMCs from cured/endemic/infected kala-azar patients, we observed ∼2.0 to 4.0 times better proliferative response as well as IFN-ã and IL-12 in comparison to SLD and low concentration of IL-10 in culture supernatants of rLdTPI -induced PBMCs from cured kala-azar patients as well as endemic contacts. It is likely that the individuals from the endemic area are mostly exposed thus exhibiting such levels of responses. As expected, rLdTPI did not induce proliferation or cytokine production in healthy subjects, suggesting its specificity toward *L. donovani* infection. The analysis of cellular immune response of the rLdTPI was further validated in hamsters’ lymphocytes/macrophages in order to correlate the observations made with the human PBMCs as the systemic infection of the hamster with *L. donovani* is very similar to human kala-azar [22]. In the absence of cytokine reagents against hamsters, we have evaluated the effect of rLdTPI on LTT and NO production by peritoneal macrophages of hamster. It is well documented that in case of leishmanial infections, macrophages become activated by IFN-γ released from parasite-specific T cells, and are able to destroy intracellular parasites through the production of several mediators, principal among which is NO [53], [54]. rLdTPI gave significantly higher cellular responses viz. LTT as well as NO release against all the cured hamsters in comparison to normal and infected ones. The limitations of this *in vitro* study based on a convenience human sampling, may not perhaps allow drawing solid conclusions regarding the immunogenicity of rLdTPI. However, since the cellular responses of the antigen in human subjects were further validated in cured hamsters, our findings therefore accentuate that *L. donovani*-primed PBMCs from cured kala-azar patients and hamsters after stimulation with rLdTPI exhibit a strong Th1-type cellular response, which should be protective in nature.

Successful immunization that induces protection against leishmaniasis is highly dependent on adjuvants or delivery system that preferentially stimulates the Th1 phenotype of immune response and plasmid DNA is one of the most interesting vaccine delivery system. In contrast to conventional immunization that results in stimulating primarily CD4-T-cell responses, DNA immunization has been shown to stimulate both CD4- and CD8-T-cell responses [55], [56], [57]. Based on this, in the present study, we carried out DNA vaccination with *LdTPI*- DNA in hamsters and challenged with the virulent strain of *L. donovani.* The immunized hamsters survived the lethal challenge and remained healthy more than 8 months, whereas all non-immunized and blank vector-immunized hamsters succumbed to the lethal *L. donovani* challenge within 3–4 mo p.c.

We further assessed whether the vaccination with *LdTPI*-DNA was able to generate immune response in hamsters since, a major factor of the immune mechanism(s) is the development of strong CMI responses like T-cell responses, NO production and DTH responses which are responsible for protection and are also supposed to contribute to healing in VL [41], [58], [59], [60]. In the present study, it was evident that all *LdTPI*-DNA vaccinated hamsters challenged with *L. donovani* have a specific active T-cell response because they displayed significant LTT response after challenge; on the other hand, this response was severely impeded in non-immunized infected and healthy control hamsters. Further, the supernatant of SLD-stimulated lymphocytes from *LdTPI*-DNA vaccinated hamsters produced a remarkable level of NO in the macrophages of naive hamsters which also supported the view regarding the up-regulation of iNOS by Th1 cell-associated cytokines and confirms that the NO-mediated macrophage effector mechanism is critical in the control of parasite replication in the animal model [61]. In addition, successful vaccination of humans and animals is often related to antigen-induced DTH responses *in vivo* and T-cell stimulation with antigen *in vitro* [22], [62], suggesting a correlation between CMI responses and immunity to infection in this model. There was a low level of parasite-specific DTH responses observed in infected and vector control animals which, on the other hand, was strongly expressed in hamsters immunized with DNA vaccine. Apart from diminished cellular responses, active VL is also associated with the production of high levels of the *Leishmania* specific antibody particularly IgG and IgG1, which are observed before detection of parasite-specific T cell response [63]. On the other hand, as a measure of CMI, the elevation of IgG2 is consistent with the development of effective immune responses [33]. The transcript of IFN-γ, a signature cytokine of the Th1-type response that has a dominant effect on macrophage microbicidal responses and other effector killing mechanisms, along with TNF-á, often reported to act in concert to activate iNOS for the production of NO [64], [65], were found to be down-regulated [58] in infected hamsters, whereas their expression was observed to be increased many fold in the immunized hamsters. We have also found an extreme down-regulation in the of IL-10, reported to be down regulate IL-12 for disease progression [66] and IL-4, considered to be a marker for Th2 response [67], in *LdTPI*-DNA vaccinated hamsters compared with infected control hamsters. The level of other Th1 cytokine-IL-12, an immune-regulatory cytokine for initiation and maintenance of the Th1 response and plays an important role in the induction of IFN-ã production by T and NK cells [68], [69], [70], was completely down-regulated in infected hamster, where as high levels of IL-12 mRNA transcripts were observed in vaccinated hamsters. However, TGF-â-a pleiotropic cytokine, having immunosuppressive properties, is also documented in leishmania disease progression [71] and known to be expressed at a moderate level even in normal hamsters [22], [41], [58], was apparently down-regulated in all the immunized hamsters throughout the experiment. Further, it has been well established that level of IgG and IgG1 antibody increases with the *L. donovani* loads [41], which however, was present at very low level in the vaccinated group and thus consistent with the decreasing parasite loads. The significant increase in the IgG2 levels in the vaccinated animals only is a phenotypic marker of enhanced CMI.

In a nutshell, our studies have for the first time indicated that LdTPI, a glycolytic enzyme, is also capable of inducing a robust cellular immune response *in vitro* against both *L. donovani*-primed lymphocytes from cured kala-azar patients and hamsters and also eliciting a strong protective response against experimental VL. Hence, the significant immunogenic and prophylactic efficacy of *LdTPI*-DNA makes it a strong and ideal prophylactic vaccine candidate.
